# Genomic Screening of Antimicrobial Resistance Markers in UK and US *Campylobacter* Isolates Highlights Stability of Resistance over an 18-Year Period

**DOI:** 10.1128/aac.01687-21

**Published:** 2022-04-11

**Authors:** Arnoud H. M. van Vliet, Siddhartha Thakur, Joaquin M. Prada, Jai W. Mehat, Roberto M. La Ragione

**Affiliations:** a School of Veterinary Medicine, Department of Pathology and Infectious Diseases, Faculty of Health and Medical Sciences, University of Surrey, Guildford, United Kingdom; b School of Veterinary Medicine, Department of Veterinary Epidemiology and Public Health, Faculty of Health and Medical Sciences, University of Surrey, Guildford, United Kingdom; c School of Biosciences and Medicine, Faculty of Health and Medical Sciences, University of Surrey, Guildford, United Kingdom; d Department of Population Health & Pathobiology, College of Veterinary Medicine, North Carolina State University, Raleigh, North Carolina, USA

**Keywords:** antimicrobial resistance, antibiotic stewardship, *Campylobacter*, surveillance, quinolones, tetracycline, aminoglycosides, macrolides, whole-genome sequencing, surveillance studies

## Abstract

Campylobacter jejuni and Campylobacter coli are important bacterial causes of human foodborne illness. Despite several years of reduced antibiotics usage in livestock production in the United Kingdom (UK) and United States (US), a high prevalence of antimicrobial resistance (AMR) persists in *Campylobacter*. Both countries have instigated genome sequencing-based surveillance programs for *Campylobacter*, and in this study, we have identified AMR genes in 32,256 C. jejuni and 8,776 C. coli publicly available genome sequences to compare the prevalence and trends of AMR in *Campylobacter* isolated in the UK and US between 2001 and 2018. AMR markers were detected in 68% of C. coli and 53% of C. jejuni isolates, with 15% of C. coli isolates being multidrug resistant (MDR), compared to only 2% of C. jejuni isolates. The prevalence of aminoglycoside, macrolide, quinolone, and tetracycline resistance remained fairly stable from 2001 to 2018 in both C. jejuni and C. coli, but statistically significant differences were observed between the UK and US. There was a statistically significant higher prevalence of aminoglycoside and tetracycline resistance for US C. coli and C. jejuni isolates and macrolide resistance for US C. coli isolates. In contrast, UK C. coli and C. jejuni isolates showed a significantly higher prevalence of quinolone resistance. Specific multilocus sequence type (MLST) clonal complexes (e.g., ST-353/464) showed >95% quinolone resistance. This large-scale comparison of AMR prevalence has shown that the prevalence of AMR remains stable for *Campylobacter* in the UK and the US. This suggests that antimicrobial stewardship and restricted antibiotic usage may help contain further expansion of AMR prevalence in *Campylobacter* but are unlikely to reduce it in the short term.

## INTRODUCTION

Antibiotics are one of the great success stories of the 20th century, changing medicine and agricultural practices (1). Unfortunately, the fairytale did not last, and over the last decades, antimicrobial resistance (AMR) has become a significant problem (2). The excessive and inappropriate use of antibiotics in human and veterinary medicine and their widespread use as growth promoters in agriculture have led to a rise of resistance to many classes of antibiotics, including last-resort, critically important antimicrobials for human medicine (3). This is now recognized as a serious and global crisis, especially given the lack of progress in developing novel antibiotics (4). The World Health Organization declared AMR a severe threat to global public health in 2014 (5) and called for the urgent introduction of multisectoral measures to prevent further development of AMR. This includes responsible use, which falls under antimicrobial stewardship (6, 7). One of the aspirations of reduced use of antimicrobials is that in the absence of antibiotic selection, susceptible isolates should have a fitness advantage over resistant isolates (8, 9), thereby restricting expansion of drug-resistant populations.

While phenotypic testing in the laboratory is still regarded as the gold standard, molecular tools such as those based on whole-genome sequencing (WGS) are strong contenders to supersede phenotypic testing (10, 11) and have been successfully used for foodborne pathogens such as Salmonella and *Campylobacter* (12–15). However, harmonization of databases and software tools is still required to avoid interlaboratory reproducibility issues (16). Tools such as the NCBI AMRFinder software and curated Bacterial Antimicrobial Resistance Reference Gene Database at NCBI will certainly assist with such standardization (17).

The bacterial pathogen *Campylobacter* is one of the leading causes of human bacterial diarrheal illness worldwide (18), and the European Food Safety Authority (EFSA) and the Centers for Disease Control and Prevention (CDC) have reported similar levels of campylobacteriosis in the European Union and United States (19, 20). The causative agents, Campylobacter jejuni and Campylobacter coli, are commonly associated with poultry, wild birds, ruminants, and pigs, with undercooked meat and cross-contamination seen as common causes of infection (21). The major category of antibiotics used to treat *Campylobacter* is macrolides, such as erythromycin in humans. In contrast, aminoglycosides such as gentamicin and streptomycin, macrolides, quinolones such as ciprofloxacin and nalidixic acid, and tetracycline have been commonly used in agricultural and veterinary settings (22–24). The WHO has listed *Campylobacter* as a high-priority antibiotic-resistant pathogen due to the rapid rise in quinolone resistance in *Campylobacter* (2).

One of the requirements for unbiased monitoring of antimicrobial resistance levels in *Campylobacter* is the existence of large-scale surveillance studies with genome sequences and matching metadata deposited in public repositories such as NCBI and EMBL. Historical isolates and collections may suffer from biases introduced by a focus on isolates with “interesting” phenotypes such as multiple antibiotic resistances. Only two countries have instigated large-scale genome sequencing-based surveillance of *Campylobacter*: the United Kingdom (UK) and United States (US). The UK surveillance has focused on clinical isolates (25, 26), whereas the US surveillance combines isolates from clinical, food, and animal sources (27, 28).

In this study, we collected the available whole-genome sequences from UK and US C. jejuni and C. coli isolates between 2001 and 2018 and have assessed the presence of genes conferring resistance to aminoglycosides, macrolides, quinolones, and tetracyclines. We highlight the trends over time and differences between the two countries, and we show the stability of the frequency of resistance to four antibiotic categories in *Campylobacter* in the UK and US, which could raise concerns about future developments.

## RESULTS

### Comparison of genome assembly- and sequence read-based AMR marker detection.

To assess the efficacy of the NCBI AMRFinder software tool with *Campylobacter* genome sequences, we compared it with a recent AMR analysis of 381 UK C. jejuni and C. coli isolates (13). Painset et al. (13) performed AMR marker detection directly on the Illumina sequencing reads using in-house scripts, whereas we used the NCBI AMRFinder software tool, which requires genomes to be assembled first. There were no false positives detected using the NCBI AMRFinder software for any of the antibiotic resistance classes (Table 1), and there was 100% agreement for detection of quinolone resistance (GyrA D87 mutation) and aminoglycoside resistance markers. Screening for tetracycline resistance resulted in 2/161 *tetO*-positive isolates that were initially reported negative for the *tetO* gene using NCBI AMRFinder. Further investigation of these two samples showed the *tetO* gene to be split over two contigs, which was detectable by secondary screening with BLAST using the Abricate software tool, and this was subsequently done as standard (see Table S1 posted at https://doi.org/10.6084/m9.figshare.19292126.v1). Finally, macrolide resistance based on 23S rRNA gene mutations resulted in three negative samples previously reported positive (13). However, all three samples contained both the wild-type and mutated 23S rRNA alleles, suggesting that only one or two of the three C. jejuni 23S rRNA genes mutated. Genome assembly algorithms are likely to ignore the minority gene variants, leading to these samples testing negative. Finally, there were five genome assemblies that contained an *aph*(3′)-IIIa aminoglycoside resistance gene (Table S1) not included in the work of Painset et al. (13). Overall, there was very good concordance between the two tests, and we considered this a validation for testing *Campylobacter* genome assemblies with the NCBI AMRFinder software tool.

**TABLE 1:** Validation of antimicrobial resistance marker detection in *Campylobacter* genomes using NCBI AMRFinder with assembled genomes, compared to the study of Painset et al. (13), which used detection of antimicrobial resistance markers with Illumina sequencing reads

Antibiotic category	No. positive/total^*a*^	No. negative/total^*a*^	% agreement (positive, negative)^*a*^
Aminoglycosides	14/14^*b*^	362/362	100, 100
Macrolides	28/28^*c*^	350/350	100, 100
Quinolones	172/172	209/209	100, 100
Tetracycline	159/161^*d*^	220/220	98.6, 100

a Detection of AMR markers using NCBI AMRFinder version 3.6.7 on genomes assembled using Shovill version 1.0.9, using samples described by Painset et al. (13). The predictions from NCBI AMRFinder were compared to the results from Painset et al. (13) and the percent agreement reported for AMR marker-positive and AMR marker-negative samples.

b Five samples contained an additional *aph*(3′)-IIIa aminoglycoside resistance gene not reported in reference 13 and were not included in the percentage calculation. Two of these samples were phenotypically resistant to aminoglycosides (13).

c Three negative samples were previously reported as erythromycin susceptible in resistance testing and showed inconclusive mutation detection in the study by Painset et al. (13). These were excluded from the percentage calculations.

d The two negative samples had the *tetO* resistance gene divided over two contigs, which resulted in NCBI AMRFinder being unable to detect the *tetO* resistance gene. Additional screening using Abricate version 0.9.8 with the “--mincov 30” setting (allowing screening for gene fragments) allowed the detection of this AMR marker (Table S1).

### Characteristics of a 2001–2018 C. jejuni and C. coli genome assembly database from public sources.

**FIG 1:**
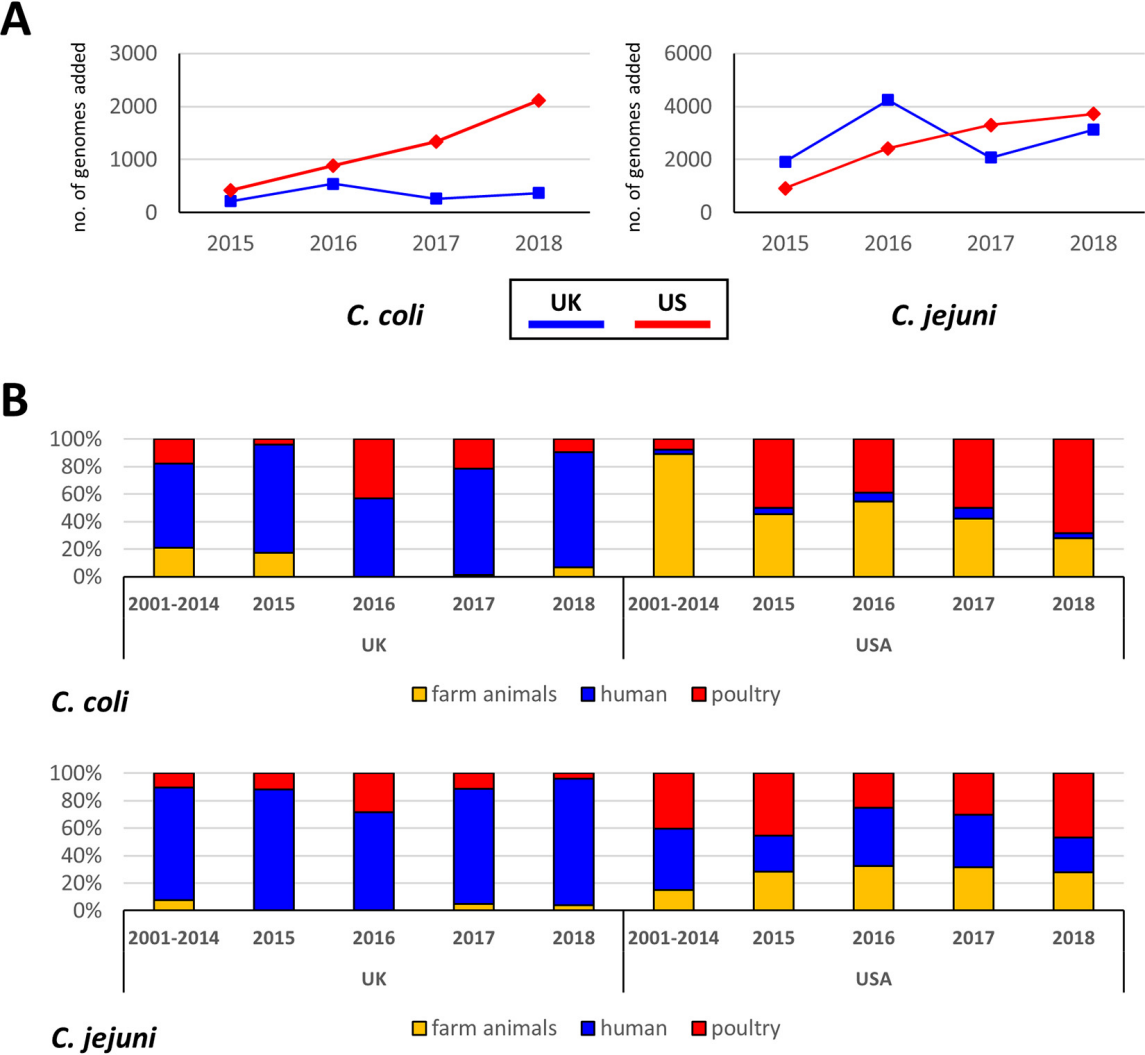
Breakdown of the UK and US C. coli and C. jejuni samples obtained in surveillance programs from 2015 to 2018. (A) Number of genome sequences per individual year from 2015 to 2018. The number of US genome sequences shows steady growth each year, whereas the number of UK genome sequences varies per year without a clear trend. (B) Comparison of the isolation source category of the UK and US samples from 2015 to 2018, with the historical isolates from 2001 to 2014 included. Within the UK samples, the human source category is dominant, consistent with the major surveillance category being the Oxfordshire sentinel surveillance (25, 26), while US isolates have a much higher contribution of farm animals (pigs and ruminants) and poultry (27, 28).

A total of 32,256 C. jejuni and 8,776 C. coli genomes were included in our study. The distribution over the year categories is shown in Fig. 1A; for C. coli, approximately 400 UK samples were added for each of the years from 2015 to 2018, whereas the number of US C. coli samples increased from approximately 500 samples in 2015 to just over 2,000 in 2018. Similar trends were observed for C. jejuni, where the number of UK samples varied between approximately 2,000 and 4,000 per year, while the number of US samples increased from approximately 1,000 in 2015 to just under 4,000 in 2018. The samples from 2001 to 2014 were combined, as these did not have consistent availability of samples, with yearly numbers varying between 0 and 350 (C. coli, UK), 7 and 704 (C. coli, US), 10 and 1,881 (C. jejuni, UK) and 0 and 277 (C. jejuni, US) (see Fig. S1 and S2 at https://doi.org/10.6084/m9.figshare.19292126.v1).

The C. jejuni and C. coli samples were split into several groups: UK- and US-derived samples, and within each, historical samples from 2001 to 2014 versus the individual years from 2015 to 2018, and the three source categories (farm animals, humans, and poultry). Comparison of these categories (Fig. 1B) showed that UK samples were dominated by human isolates (approximately 70% for C. coli, versus 5% for C. coli from the US), with farm animal samples poorly represented. In contrast, US samples showed a better representation of farm animal samples for both C. jejuni and C. coli, especially in samples derived from 2001 to 2014.

### Distribution of antimicrobial resistance markers in C. jejuni and C. coli.

The antimicrobial resistance profiles were determined for all the C. jejuni and C. coli genomes using NCBI AMRFinder. The presence of any of the 44 resistance markers was translated into aminoglycoside resistance (20 markers), macrolide resistance (9 markers), quinolone resistance (10 markers), or tetracycline resistance (5 markers). Table 2 shows the breakdown of the number of isolates predicted to be resistant for both C. coli and C. jejuni.

**TABLE 2:** Distribution of antibiotic resistance classes in C. coli and C. jejuni genome sequences (2001 to 2018 combined)

Organism (no. of isolates)	No. (%) of isolates with indicated no. of antibiotic resistance classes								
	0	1^*a*^	2^*b*^	3 (MDR)^*c*^	4 (MDR)	Aminoglycosides	Macrolides	Quinolones	Tetracycline
C. coli (8,776)	2,828 (32.2)	2,079 (23.7)	2,551 (29.1)	1,146 (13.1)	172 (2.0)	3,105 (35.4)	845 (9.6)	2,520 (28.7)	4,837 (55.1)
C. jejuni (32,256)	15,044 (46.6)	8,749 (27.1)	7,647 (23.7)	718 (2.2)	98 (0.3)	1,770 (5.5)	261 (0.8)	10,276 (31.9)	14,283 (44.3)

a The majority of isolates were aminoglycoside resistant (C. coli, 17%), quinolone resistant (C. coli, 26%; C. jejuni, 31%), or tetracycline resistant (C. coli, 54%; C. jejuni, 67%).

b The majority of isolates were aminoglycoside plus tetracycline resistant (C. coli, 55%; C. jejuni, 11%) or quinolone plus tetracycline resistant (C. coli, 34%; C. jejuni, 88%).

c The majority of isolates were aminoglycoside plus macrolide plus tetracycline resistant (C. coli only, 28%) or aminoglycoside plus quinolone plus tetracycline resistant (C. coli, 64%; C. jejuni, 86%). MDR, multidrug resistant.

For C. coli, 15.1% of samples were predicted to be multidrug resistant (MDR; resistant to three or more classes of antibiotics), whereas only 2.5% of C. jejuni isolates were predicted to be MDR. Similarly, almost half of C. jejuni samples (46.6%) did not have any resistance marker, compared to one-third of C. coli samples (32.2%). Approximately half of the C. coli and C. jejuni isolates were predicted to be resistant to tetracycline (55.1% and 44.3%, respectively), while about one-third of C. coli and C. jejuni isolates were predicted to be resistant to quinolones (28.7% and 31.9%, respectively). Notable differences were seen in aminoglycoside resistance (C. coli, 35.4%, versus C. jejuni, 5.5%) and macrolide resistance (C. coli, 9.6%, versus C. jejuni, 0.8%) (Table 2). Of note, the prevalences of gentamicin resistance were 213/8,776 (2.4%) for C. coli and 127/32,256 (0.4%) for C. jejuni, with most gentamicin resistance markers detected in US samples (see Table S1 at https://doi.org/10.6084/m9.figshare.19292126.v1).

### Comparison of AMR levels between the UK and US (2001 to 2018).

**FIG 2:**
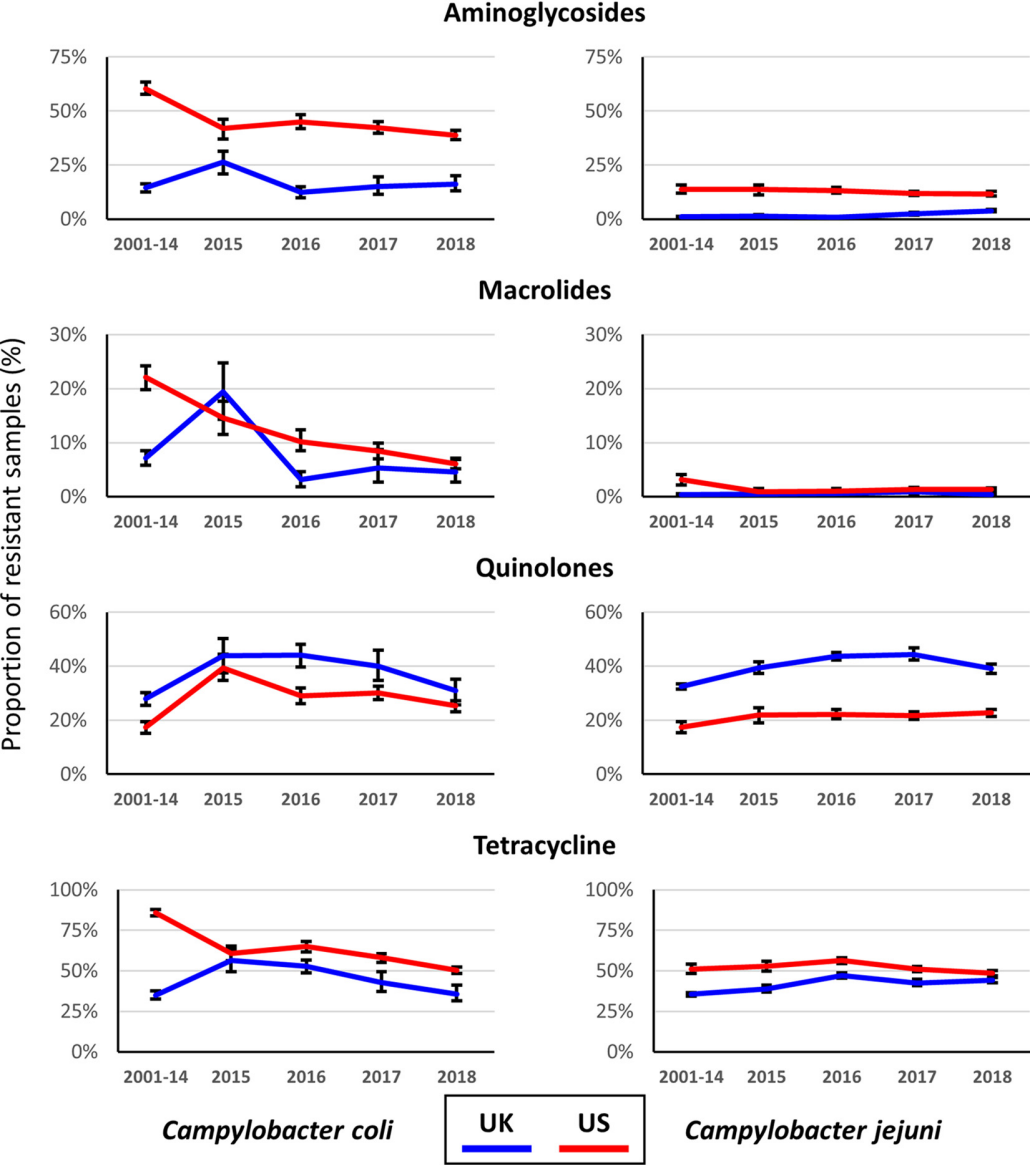
Comparison of the proportion of UK and US C. coli and C. jejuni samples resistant to aminoglycosides, macrolides, quinolones, and tetracycline for individual years from 2015 to 2018, with the average of the period from 2001 to 2014 provided for comparative purposes. Error bars show the 2.5% and 97.5% quantiles, based on 500 bootstraps.

The UK and US are currently the two countries which do large-scale genome sequencing-based surveillance of *Campylobacter* (14, 26), and this allows for insight in the dynamics of antibiotic resistance in these two industrialized countries over time. The years 2015 to 2018 were used individually while combining the samples from 2001 to 2014 to function as a possible baseline for comparative purposes. The predicted resistances to aminoglycosides, macrolides, quinolones, and tetracycline were plotted out for the UK and US isolates and compared for trends (Fig. 2), with the individual years from 2001 to 2018 shown in Fig. S1 and S2 at https://doi.org/10.6084/m9.figshare.19292126.v1.

The general trend for both C. coli and C. jejuni was that the proportion of aminoglycoside- and tetracycline-resistant isolates was significantly higher in the US than in the UK between 2015 and 2018, while the prevalence of quinolone resistance was significantly lower in the US (Fig. 2), prevalences of macrolide resistance were similar for both countries for C. jejuni, and the prevalence was higher for US C. coli for three of the four years but lower in 2015 (Fig. 2). Approximately 40% of the US C. coli isolates were predicted to be resistant to aminoglycosides (range, 39 to 45%), whereas in the UK C. coli isolates, the value was approximately 20% (range, 12 to 26%). For C. jejuni, the prevalence of aminoglycoside resistance was between 12 and 14% for the US isolates, compared to 1 to 4% for the UK isolates. Differences were less pronounced for tetracycline resistance for C. jejuni (range, 39 to 46% for UK samples, versus 48 to 56% for US samples), while in C. coli this varied between 36 and 56% for the UK isolates and between 50 and 65% for the US isolates. Macrolide resistance was present in much higher levels in C. coli in both the UK and the US isolates, ranging from 3 to 19% in the UK isolates and 6 to 14% in the US C. coli isolates, while it was between 0 and 1% in the UK and the US C. jejuni isolates. Finally, the trend was reversed for quinolone resistance, with a higher proportion of the UK isolates predicted to be quinolone resistant. For C. coli, the proportions of quinolone-resistant isolates were 31 to 44% for UK isolates and 25 to 39% for US isolates, while for C. jejuni the proportions were 39 to 44% for UK isolates and 22 to 23% for US isolates. For each of the *Campylobacter* species and antibiotic class, statistically significant differences were generally observed between countries and year (see Table S2 at https://doi.org/10.6084/m9.figshare.19292126.v1).

Besides, the differences between the two countries, we also observed that the only antibiotic class for which there was a clear decrease in resistance from 2015 to 2018 was macrolides (Fig. 2), with decreases from 15% to 6% in the US isolates and from 19% to 5% in the UK isolates. For the other three antibiotic classes, there was little change from 2015 to 2018, and especially in C. coli, the proportions of resistant isolates remained high for aminoglycosides (US), quinolones (UK and US), and tetracycline (UK and US). For C. jejuni, the levels of quinolone resistance are especially of concern in the UK.

### Associations between isolation source and multilocus sequence type (MLST) genotypes and AMR markers in C. coli and C. jejuni.

**FIG 3:**
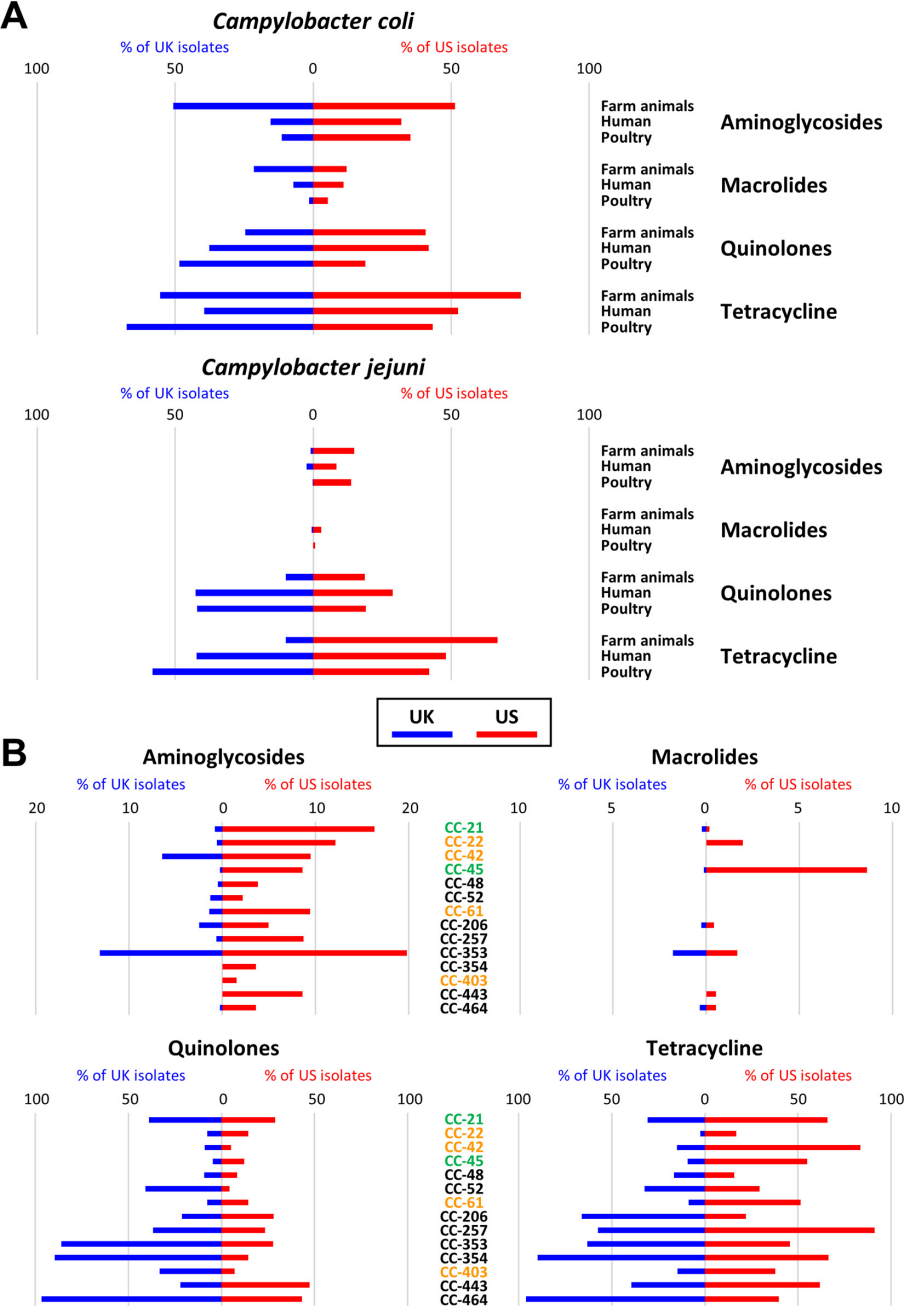
Tornado plots displaying the relative contributions of the source categories (A) and C. jejuni MLST clonal complexes (B) to the individual antibiotic classes (aminoglycosides, macrolides, quinolones, and tetracycline). The horizontal axis shows the percentage UK samples from 100 to 0 on the left half and the percentage of US samples from 0 to 100 on the right half for each of the categories. Only the samples from the years 2015 to 2018 are included.

Due to the pronounced differences in isolation source between the UK and US (Fig. 1B), we investigated whether there were differential contributions to the proportions of antibiotic-resistant isolates for C. coli and C. jejuni isolates from 2015 to 2018 (Fig. 3A). There was no clear pattern of overrepresentation of any isolation source for the AMR isolates in C. coli. However, the low number of farm animal isolates from the UK may hide some effects. Similarly, for C. jejuni there was no clear link with any of the three source categories (Fig. 3A).

We also investigated whether specific multilocus sequence type (MLST) clonal complexes (CC) were associated with specific antibiotic resistances in the UK and US isolates. For C. coli, 97.3% of the isolates are from CC-828. For C. jejuni, we looked at the clonal complexes for which for both the UK and US there were at least >100 samples available for 2015 to 2018 (Fig. 3B). For aminoglycoside resistance, there was a spread over all the major clonal complexes for US isolates, with CC-21 and CC-353 having the highest proportion of resistant isolates (17 to 20%). In contrast, for the UK isolates, aminoglycoside resistance was primarily found in CC-353 and CC-42. The CC-353 clonal complex was also involved in macrolide resistance, although this played a minor role in the C. jejuni isolates, with CC-45 showing the highest proportion of resistant isolates in the United States. For both quinolone resistance and tetracycline resistance, all major MLST genotypes contributed to resistance, but the major contribution to quinolone resistance in UK isolates came from CC-353, CC-354, and CC-464, where almost all isolates were predicted to be quinolone resistant, consistent with earlier reports (26, 29). Finally, the highest proportions of tetracycline resistance were associated with CC-354 and CC-464 for UK isolates and CC-257 and CC-42 for US isolates. Most of the clonal complexes reported here were primarily associated with poultry and human infections, except for CC-42, associated with cattle.

## DISCUSSION

In this study, we have exploited the possibilities afforded by the active genome sequencing-based surveillance for *Campylobacter* in the UK and US, to determine the trends in AMR over recent years and compare these trends within and between the two countries. Similar studies have previously been conducted in individual countries, but often at a smaller scale than employed in this study or focusing on a shorter period. For instance, a recent UK study investigated 528 human isolates from 2015 to 2016 (13), while a 2018 US-based study focused on 589 isolates from 2015 (14). These and other genome sequencing-based studies have all shown that identifying resistance markers in genome sequences matches well with phenotypic resistance. Hence, we are confident that our study appropriately represents AMR trends detected in 32,256 C. jejuni and 8,876 C. coli samples, as listed in Table S1 at https://doi.org/10.6084/m9.figshare.19292126.v1.

Monitoring of AMR on this scale requires active surveillance, with sequencing data and metadata being released in the public domain. We have combined the information present in the GenBank database, which contains both US and UK samples, and the *Campylobacter* PubMLST database, which includes a large number of UK isolates. Our data set is based on a deduplicated, quality-controlled set of genome sequences with strict requirements for available metadata. The criteria for isolate source, year, and country data allowed direct comparison between the UK and US *Campylobacter* samples for C. coli and C. jejuni (Fig. 1B). The UK data set leans heavily on the Oxfordshire Sentinel Surveillance (25, 26), which explains the dominance of human samples in the UK data set in each individual year. This contrasts with the US data set, which has an equal distribution over the three source categories. While this is not ideal for comparative purposes, we do note that for C. jejuni the distribution over the dominant MLST clonal complexes was very similar (Fig. 3), with the major differences being that CC-257 and CC-464 were prevalent in higher proportions in UK samples (5% and 6% more of the total number of samples). In comparison, CC-353 was more prevalent in US samples (7% more). All three clonal complexes are primarily associated with poultry, and hence, for C. jejuni, we concluded that the data sets could still be compared. For C. coli, this was more difficult to assess, as most samples clustered in the agricultural clade 1A (30, 31), which contains only CC-828 and a minority of samples not part of this clonal complex. The individual MLSTs may primarily represent the STs prevalent in the two countries and may be affected by the differences in isolation sources between the samples from the two countries (Fig. 1B).

In this study, we have focused on four classes of antibiotics, due to their relevance in *Campylobacter* and their usage in the categories of hosts investigated. The macrolide erythromycin and the fluoroquinolone ciprofloxacin are used to treat human infections with *Campylobacter* (22). In contrast, all four classes of antibiotics are used therapeutically in the veterinary sector for livestock, albeit not for control of *Campylobacter*, as the usage is associated with other infectious diseases in livestock (32). As highlighted earlier, the WHO has listed *Campylobacter* as a high-priority antibiotic-resistant pathogen (2), especially linked to fluoroquinolone resistance in *Campylobacter*. Antimicrobial resistance in *Campylobacter* spp. is a combination of *de novo* point mutations associated with quinolone and macrolide resistance and genetic exchange on plasmids and other mobile elements for aminoglycoside and tetracycline resistance genes (33). In addition, the natural competence of C. jejuni and C. coli (34, 35) may allow further dissemination of resistance through populations. Although the usage of antibiotics has been limited in the UK and the US for a considerable period of time (32, 36, 37), there was no apparent reduction visible in the prevalence of resistance in the large numbers of C. jejuni and C. coli samples investigated in this study (Fig. 2). Our statistical analyses suggest that there may be small increases in C. jejuni resistance to some antibiotics in recent years, compared to the levels of 2014 and earlier (see Table S2 at https://doi.org/10.6084/m9.figshare.19292126.v1). However, due to the composition of the data set and its retrospective nature, this is something that requires a prospective study to further investigate, which falls outside the aims and scope of the work presented here. A possible hypothesis for the observations is that resistance to especially tetracyclines, aminoglycosides, and quinolones is not detrimental to *Campylobacter* or not sufficiently detrimental to allow outcompetition by susceptible isolates. Conversely, resistance may even be associated with advantageous phenotypes, as the gyrase mutations associated with quinolone resistance gave rise to increases in virulence phenotypes in C. jejuni (38, 39), while *tetO*-containing plasmids are ubiquitous and stable in C. jejuni and C. coli (40, 41). Aminoglycoside resistance genes are especially widespread in C. coli, and their association with mobile elements (33, 42) may assist their stability in the populations.

The similarities between the UK and US data sets suggest that the situation in other industrialized countries may be similar, but this will require more intensive surveillance programs and will need to balance clinical, food, and agricultural samples, including sewage and water sources. A recent study reported that fluoroquinolone usage in pigs and poultry in France from 2011 to 2018 decreased by >70% in poultry and almost 90% in pigs. However, resistance to ciprofloxacin increased in poultry *Campylobacter* isolates from 50% to approximately 60%, whereas in Escherichia coli, prevalence of ciprofloxacin resistance was stable in broiler and pig isolates but decreased in turkey isolates (43). Similar studies have been done in other countries but often are based on small or limited data sets where selection bias may affect the results.

The lack of reduction of the proportion of AMR isolates in *Campylobacter* from both countries does suggest that reduced usage of antimicrobials, such as mandated by antimicrobial stewardship, will not be sufficient to reduce the incidence of AMR in *Campylobacter*, although it may well contribute positively to reining in further increases of AMR. For clinical purposes, macrolides such as erythromycin are still useful for treating C. jejuni infections, but their efficacy may be reduced for C. coli. In contrast, the high level of quinolone resistance in both C. jejuni and C. coli does not bode well for the future efficacy of ciprofloxacin treatment of *CampylobacterCampylobacter* infections in humans. With regard to aminoglycosides and tetracyclines, the latter is not recommended due to the high level of resistance, whereas with aminoglycosides, gentamicin can still be used for now, as prevalence of gentamicin resistance is still low.

Taken together, the data presented here strongly suggest that reduced usage of antibiotics has not resulted in a significant reduction of antimicrobial resistance in *Campylobacter*, which is of considerable public health and economic concern. Changes in agricultural practices, slaughter, and retail will need to be substantial to reduce the overall prevalence of *Campylobacter*. These efforts should lessen the need for antibiotic usage to achieve the goals of antimicrobial stewardship. Similar comparative studies could be done in other countries and for other foodborne zoonotic infections to assess whether this situation is unique for *Campylobacter* or mirrored in other pathogenic bacteria.

## MATERIALS AND METHODS

### *Campylobacter* genome assemblies and metadata categories included in this study.

A total of 44,751 C. jejuni and 12,709 C. coli genome assemblies were collected from the GenBank and *Campylobacter* PubMLST databases and were coupled to metadata from the GenBank files and from PubMLST. Genome assemblies were obtained from the NCBI database using ncbi-genome-download version 0.2.11 (https://github.com/kblin/ncbi-genome-download/) and supplemented with genome sequences from the *Campylobacter* PubMLST website (http://pubmlst.org/campylobacter/) (44). Metadata were extracted from GenBank flat files and the NCBI Pathogens database (https://www.ncbi.nlm.nih.gov/pathogens). All genome assemblies were screened for assembly statistics using Quast version 4.5 (45), and genome assemblies were excluded if failing two or more of the following criteria: number of contigs ≤ 200, *N*_50_ ≥ 25 kb, *L*_50_ ≤ 25 contigs, largest contig ≥ 50 kb, or number of Ns per 100 kb ≥ 50. Genomes with a total size outside 1.4 Mbp and 2.1 Mbp were automatically excluded. Duplicate entries were removed by comparing sample names, assembly statistics such as *N*_50_, *L*_50_, and genome size and metadata such as year, source, and country. After deduplication, samples were subsequently only included if the following metadata were available: isolation source, year of isolation, and isolation in the UK or US. Isolation sources were combined to give three main categories: farm animals (pigs, cattle, sheep, and goats, including milk and meat samples), human (clinical isolates), and poultry (chicken and turkey, including meat samples). Samples representing environmental, farm, generic “food,” and wild bird samples were excluded, as were samples lacking other metadata. This resulted in the exclusion of 6,327 C. jejuni and 877 C. coli samples with missing information on country, year, or isolation source; 1,634 C. jejuni and 142 C. coli samples were not from the UK or US, 3,116 C. jejuni and 2,086 C. coli samples were from outside the period from 2001 to 2018, and 1,418 C. jejuni and 828 C. coli samples were not from animal, ruminant, poultry, or human sources. We focused on 2015 to 2018 as individual years because both the UK and US were doing surveillance projects, while historical samples were categorized as 2001 to 2014. Overall, we analyzed a total of 41,032 genome sequences, represented by 32,256 C. jejuni and 8,776 C. coli genomes (see Table S1 at https://doi.org/10.6084/m9.figshare.19292126.v1).

### Screening for antimicrobial resistance markers.

Genome assemblies were screened for AMR markers using the NCBI AMRFinder software tool version 3.1.1b (17), with the October 2019 database, and the nucleotide setting (-n) and the organism switch (-O *Campylobacter*), which includes screening for point mutation-based resistances. Genome assemblies were also screened using Abricate version 0.9.8 (https://github.com/tseemann/abricate/) and the NCBI database, which screens for AMR genes but not point mutation-based resistances. Resistance genes were categorized into four types of resistance: aminoglycoside resistance [*aph*(2″)-Ia, *aph*(2″)-If, *aph*(2″)-If2, *aph*(2″)-Ig, *aph*(2″)-Ih, *aph*(2″)-IIa, *aph*(2″)-IIIa, *aph*(2″)-IVa, *apmA*, *aac*(6′)-Ie, *aac*(6′)-Im, *aad9*, *aadE*, *aadE*-Cc, *ant*(6)-Ia, *aph*(3′)-IIa, *aph*(3″)-Ib, *aph*(3′)-IIIa, *aph*(3′)-VIIa, and spw], macrolide resistance [23S_A2074C, 23S_A2074G, 23S_A2074T, 23S_A2075G, *cfr*(C), *erm*(36), *erm*(B), *erm*(C), and *erm*(F)], quinolone resistance (GyrA_D90N, GyrA_D90Y, GyrA_P104S, GyrA_T86A, GyrA_T86I, GyrA_T86K, GyrA_T86V, *qnrB*, *qnrD*, and *qnrD1*), and tetracycline resistance [*tet*(32), *tet*(L), *tet*(O), *tet*(W), and *tet*(X)]. Samples were considered multidrug resistant (MDR) when containing resistance markers for three or more classes of antibiotics.

### Comparison of genome assembly-based screening for AMR genes with sequencing read-based screening.

The supplementary data presented by Painset et al. (13) contained 381 accession numbers to FASTQ read files of UK *Campylobacter* samples used for screening for antimicrobial resistance genes. The FASTQ files were downloaded from the Sequence Read Archive using fastq-dump from the SRA toolkit (https://github.com/ncbi/sra-tools), and genomes were assembled using Spades version 3.14 (46) via the Shovill version 1.0.9 tool and standard settings (https://github.com/tseemann/shovill/). All genomes passed the Quast quality control (QC) and were screened using the NCBI AMRFinder tool version 3.6.7 (17) with the nucleotide setting (-n) and the organism switch (-O *Campylobacter*) as described above. All genome assemblies were also screened using Abricate version 0.9.8 (https://github.com/tseemann/abricate/) and the NCBI database with a minimum coverage of 30% (--mincov 30) to check for the *tetO* tetracycline resistance gene being split over two or more contigs. These 381 genome assemblies have not been included in the other analyses presented here.

### Statistical analysis of association between resistance markers and descriptor variables (country, year, and isolation source).

The presence or absence of resistance for each of the four classes of antibiotics was assessed using a generalized linear model with a logit link function (binomial family). Country, year, and isolation source of each sample were considered explanatory variables. Interactions between predictors were not considered. Bootstrapping with 500 repeats was carried out to estimate the 95% confidence interval (CI) of the prevalence of resistance for each country and year. All statistical analyses were carried out in R (version 4.0.3) (47), and the data are presented in Table S2 at https://doi.org/10.6084/m9.figshare.19292126.v1.

### Data availability.

All genome sequences used in this study are available from the GenBank/EMBL/DDBJ databases or the *Campylobacter* PubMLST website (https://pubmlst.org/organisms/Campylobacter-jejunicoli/). The assembly accession numbers (NCBI Genome) or genome identifier (ID) numbers (*Campylobacter* PubMLST) are listed in Table S1 at https://doi.org/10.6084/m9.figshare.19292126.v1, together with the metadata used and the AMR gene data.
